# The factory, the antenna and the scaffold: the three-way interplay between the Golgi, cilium and extracellular matrix underlying tissue function

**DOI:** 10.1242/bio.059719

**Published:** 2023-02-21

**Authors:** Nicola L. Stevenson

**Affiliations:** Cell Biology Laboratories, School of Biochemistry, Faculty of Biomedical Sciences University of Bristol, Biomedical Sciences Building, University Walk, Bristol BS8 1TD, UK

**Keywords:** Ciliopathies, Cilium, Extracellular matrix, Golgi, Golgin, Tissue

## Abstract

The growth and development of healthy tissues is dependent on the construction of a highly specialised extracellular matrix (ECM) to provide support for cell growth and migration and to determine the biomechanical properties of the tissue. These scaffolds are composed of extensively glycosylated proteins which are secreted and assembled into well-ordered structures that can hydrate, mineralise, and store growth factors as required. The proteolytic processing and glycosylation of ECM components is vital to their function. These modifications are under the control of the Golgi apparatus, an intracellular factory hosting spatially organised, protein-modifying enzymes. Regulation also requires a cellular antenna, the cilium, which integrates extracellular growth signals and mechanical cues to inform ECM production. Consequently, mutations in either Golgi or ciliary genes frequently lead to connective tissue disorders. The individual importance of each of these organelles to ECM function is well-studied. However, emerging evidence points towards a more tightly linked system of interdependence between the Golgi, cilium and ECM. This review examines how the interplay between all three compartments underpins healthy tissue. As an example, it will look at several members of the golgin family of Golgi-resident proteins whose loss is detrimental to connective tissue function. This perspective will be important for many future studies looking to dissect the cause and effect of mutations impacting tissue integrity.

## Teaser

**Summary:** This review aims to consider the reciprocal relationship between the Golgi, cilium and ECM in tissue biology and highlights the need for more integrated studies in this field.

## The premise

Connective tissue is one of the most abundant forms of animal tissue. It is also highly diverse, ranging from stiff, mineralised bone to elastic and pliant tendons. Such diversity is generated through the secretion of highly specialised extracellular matrices (ECMs) by dedicated cells such as fibroblasts, chondrocytes (cartilage-specific), and osteoblasts (bone-specific). Loosely packed within the tissues, these cells monitor growth signals and mechanical cues to tailor the production of ECM constituents and generate the desired biomechanical structure. Such complex and reactive regulation requires the co-ordination of numerous processes within the tissue. However, studies on ECM biology tend to focus on just one pathway at a time. Integrated studies examining the relationship between multiple points of regulation will be required to truly understand this system and move the field forward.

The primary cilium plays a key role in the regulation of ECM gene expression in response to environmental cues, while the Golgi apparatus is vital for the modification and processing of ECM proteins. The individual contribution of each of these organelles to ECM regulation is widely discussed in the literature, however there is significant evidence to suggest that their roles should not be examined in isolation. The loss of function of many Golgi- or cilium-resident proteins impacts both organelles and the ECM simultaneously. Moreover, there is considerable overlap between the disease pathology of some ciliopathies and Golgiopathies, particularly with respect to skeletal dysplasia (Reiter and Leroux, 2017; Hellicar et al., 2022; Masson and El Ghouzzi, 2022). In the spirit of integrated studies, this review aims to examine the reciprocal relationships between the ECM, Golgi and cilium, using the golgins as an example of where these intersect.

## Introducing the three compartments

### The ECM

The ECM is a complex hydrated network of proteins and carbohydrates that acts as a scaffold for cellular growth and migration, signalling molecule storage, and tissue biomechanics. In vertebrates, collagens are the most abundant ECM constituent (Box 1), however the matrisome consists of at least 300 different proteins (Naba et al., 2016). Fibrillar collagens are particularly important for providing mechanical strength and mediating cell-ECM adhesion, and their organisation is crucial to function. In tendons, collagen fibres are aligned in parallel bundles to provide tensile strength (Provenzano and Vanderby, 2006), whilst in the cornea they adopt a plywood-like arrangement of perpendicular layers of parallel fibres for transparency (Meek, 2009). Biomechanically important fibrillar networks are also formed by proteins such as fibronectin, elastins, and laminins. Meanwhile, proteoglycans promote tissue hydration, regulate collagen assembly (Chen et al., 2020), and interact with signalling molecules like TGFβ (Burton-Wurster et al., 2003). The complexities of ECM structure are exemplified using articular cartilage in Box 1.

**Box 1. The complexity of the extracellular matrix.**

(A) Composition: collagen fibres form the structural basis of all connective tissue. Collagen type I is the primary fibrillar collagen in bone, tendon and skin, while collagen type II is found in cartilage. Cell-surface receptors such as syndecans and integrins bind to collagen to mediate cell-matrix attachment. Other fibrillar proteins include elastins, which provide recoil to the tissue, and fibronectin, which further mediates cell attachment. The interstitial space is filled with proteoglycans such as aggrecan, small leucine-rich proteoglycans and hyaluronan. These form a hydrated gel to support tissue structure and are biologically active with respect to ECM organisation and signalling (Frantz et al., 2010). (B) Fibrillogenesis: fibrillar procollagen molecules are synthesised as three polypeptide chains that co-assemble to form a triple helix flanked by globular N- and C-propeptides. During secretion, these propeptide domains are cleaved to induce the end-to-end assembly of triple helices into fibrils. Fibrils are then crosslinked into fibres in the extracellular space (Canty and Kadler, 2005). (C) Cartilage: articular cartilage is an exemplar of the intricacies of ECM organisation. Progressing through the cartilage, collagen fibres align parallel to the joint surface in the superficial zone, randomly orientate in the mid-zone, and lie perpendicular to the joint in the deep zone. (D) Cartilage ECM composition is also organised locally to meet biomechanical need. The pericellular matrix (PCM) must absorb compressive forces and sheer stress and so is rich in hydrated proteoglycans. Here collagen type XI anchors cells to the ECM whilst nidogen, laminins and collagen type IX maintain chondrocyte viability. Surrounding this structure is the territorial matrix composed of a weave of collagen type II fibrils and chondroitin-sulphate rich proteoglycans which protect chondrocytes against mechanical stress. This, in turn, is surrounded by a complex fibrillar ECM which establishes the biomechanics of the tissue (Reviewed in Gilbert et al., 2021).
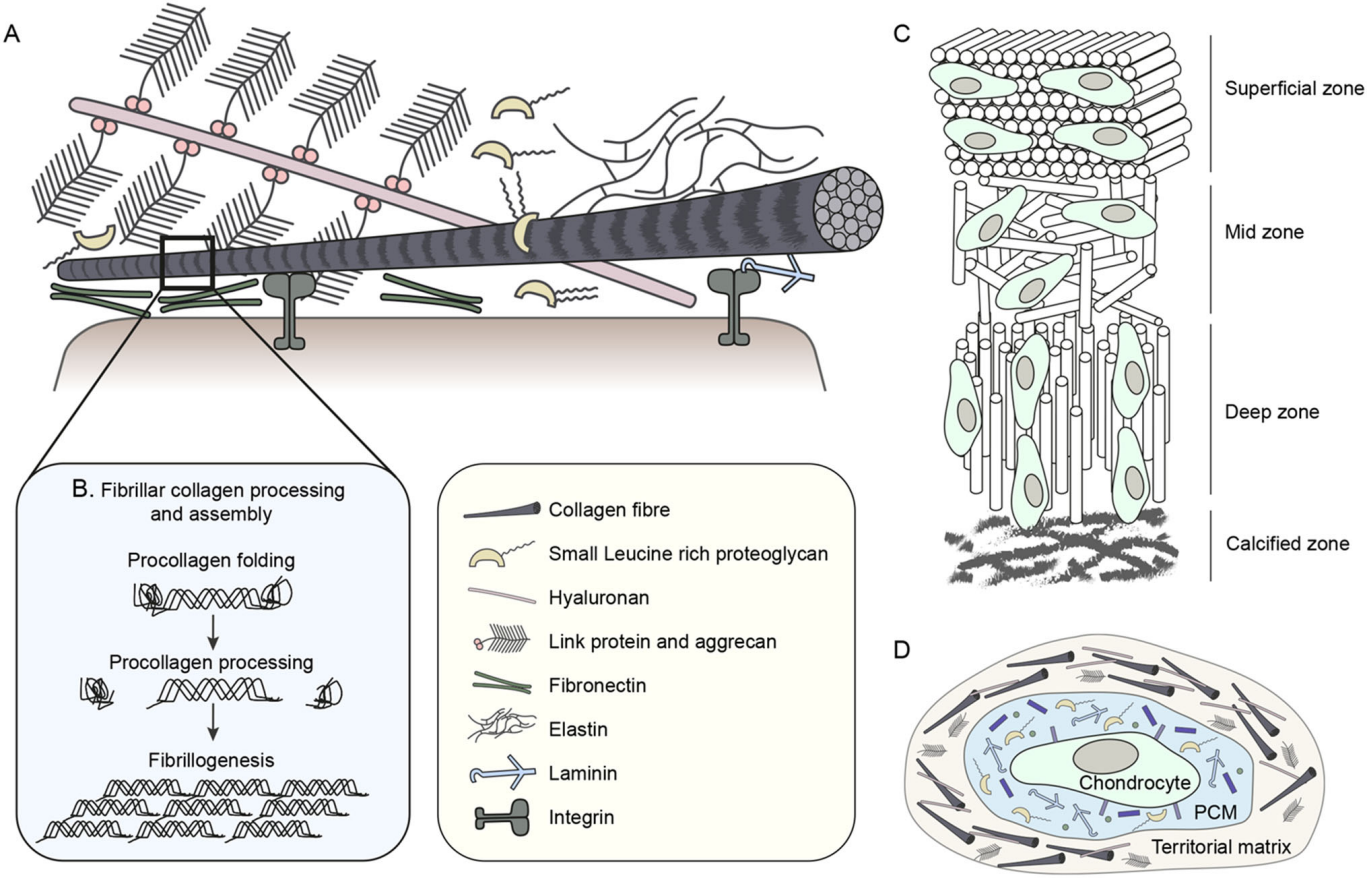

### The Golgi

**Fig. 1.**
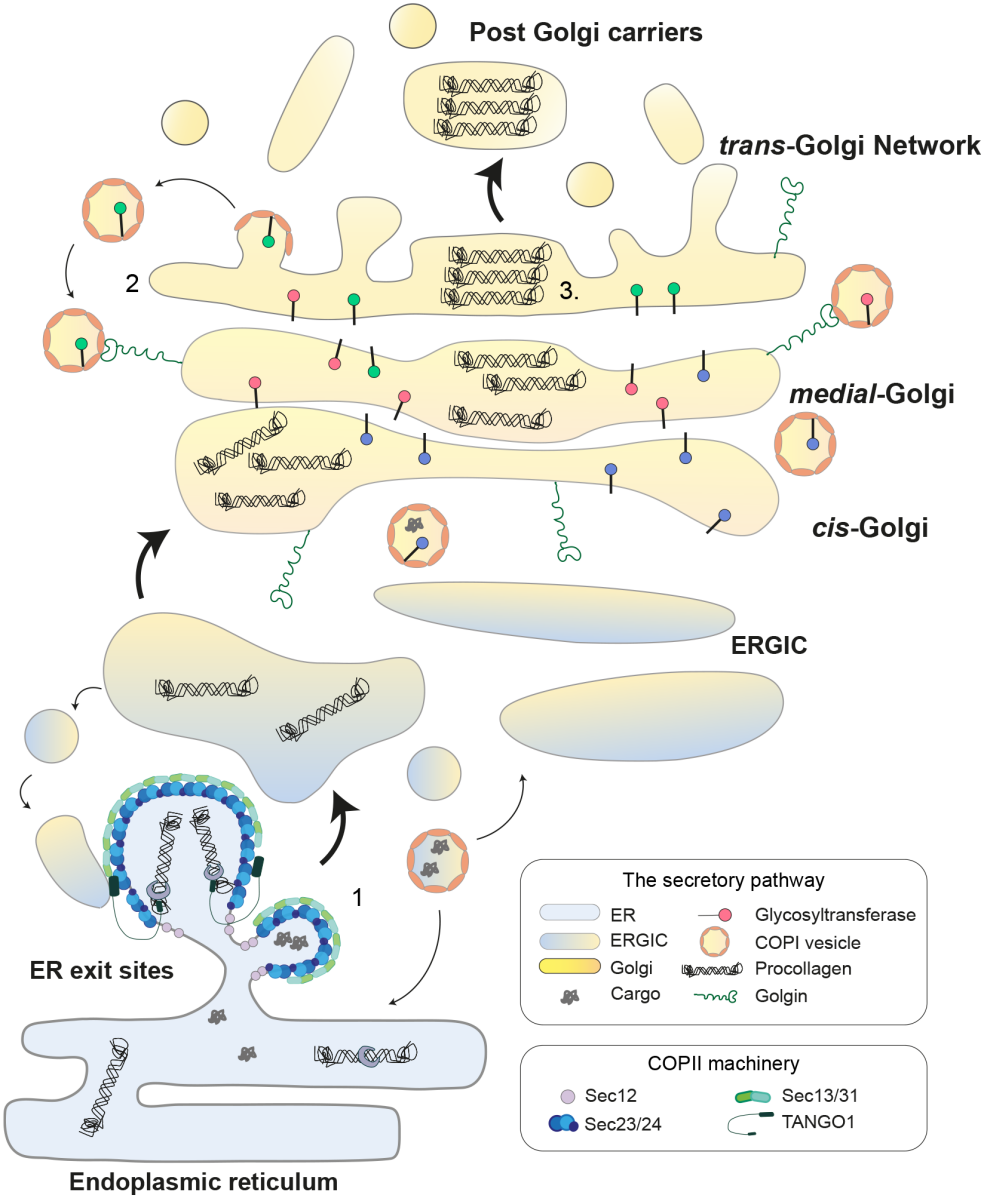
**Golgi organisation.** The Golgi exists as a stack of flattened membranes exhibiting a *cis-trans* polarity. (1) Cargo arrives at the *cis-*face courtesy of COPII-mediated transport from the ER. This is initiated at ER exit sites (ERES) by Sec12, which activates Sar1 GTPase to recruit the inner COPII coat components Sec23/24. These recruit cargo and the outer COPII coat complex, Sec13/31, to drive membrane deformation and the formation of anterograde transport carriers. Carriers then coalesce to form the ER-Golgi intermediate compartment (ERGIC) and fuse with the *cis*-Golgi. Collagen molecules are recruited to ERES by TANGO1 (McCaughey and Stephens, 2019). The heptameric coatomer complex, COPI also supports the bidirectional trafficking of vesicles between the ER and Golgi (Schroter et al., 2016; Stephens et al., 2000) (2) Transport through the Golgi proceeds by cisternal maturation in which *cis*-cisternae slowly adopt a more *trans*-cisternal identity through the retrograde recycling of enzymes. The formation of recycling vesicles is orchestrated by COPI, while their delivery is determined by tethers like the golgins (Glick and Luini, 2011). (3) Observations of collagen transport strongly support this model. Fibrillar procollagen molecules are too large to enter inter-cisternal vesicles and so must remain within the cisternae. Here they progressively organise within distended subdomains of the cisternal lumen and align themselves ready for fibrillogenesis (Bonfanti et al., 1998).

ECM molecules are synthesised within the secretory pathway, at the centre of which sits the Golgi apparatus. This organelle functions as a processing factory to facilitate the modification and sorting of almost all secreted proteins (see Fig. 1). It is composed of a polarised stack of flattened membranous compartments called cisternae, which each contain a distinct but overlapping cohort of protein modifying enzymes such as glycosyltransferases (Fig. 1; Rabouille et al., 1995). This organisation ensures that cargoes, travelling in a *cis-trans* direction through the stack, encounter Golgi enzymes in the required order (Jaiman and Thattai, 2020).

Historically, the primary mechanism of anterograde cargo transport through the Golgi has been controversial but the current consensus favours cisternal maturation (Fig. 1; Glick and Luini, 2011). In this model, cargo enters the *cis*-most cisternae and then stays within this membrane as the enzymatic and lipid content is adjusted to reflect increasingly more *trans*-cisternal identity. This process relies on the retrograde recycling of membrane and Golgi enzymes between cisternae in COPI coated vesicles (Bethune et al., 2006). Meanwhile, cargo is delivered to the *cis*-Golgi from the ER in membranous carriers generated by a COPII coat (Fig. 1; McCaughey and Stephens, 2018). Delivery of vesicular cargo to specific Golgi cisternae is dependent on hetero-oligomeric complexes such as the COG complex (Shestakova et al., 2006) and on the golgin family of coiled-coil tethers which capture incoming vesicles (Wong et al., 2017).

### Cilia

**Fig. 2.**
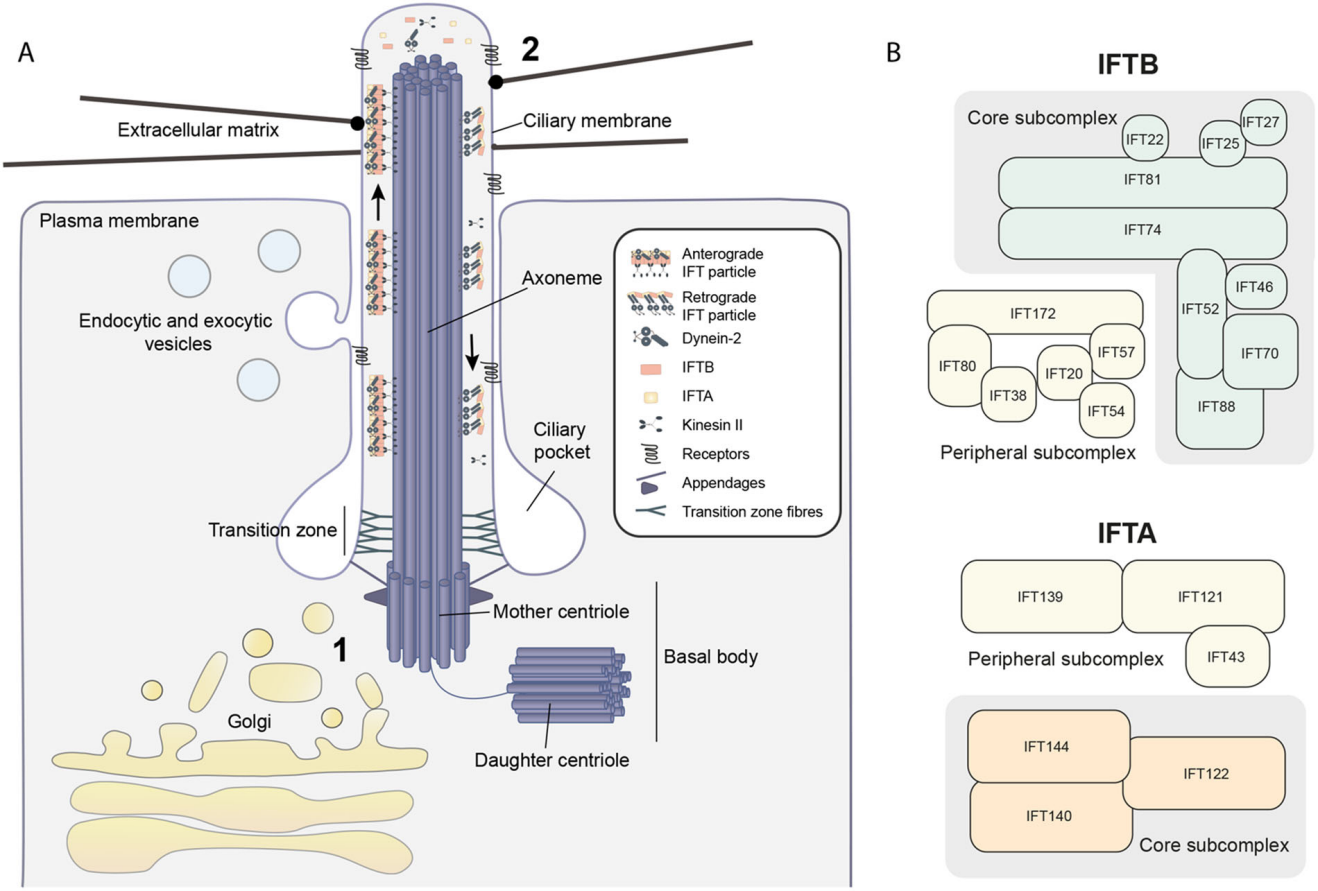
**The structure of the cilium.** (A) The structure of the primary cilium is composed of the microtubule-based axoneme, the ciliary membrane, and the basal body. The basal body is a structural unit composed of a specialised mother centriole and distal appendages linking the centriole to the ciliary membrane (Satir et al., 2010). A transition zone, composed of large protein complexes, creates a selective diffusion barrier that maintains cilium composition (Goncalves and Pelletier, 2017). Movement of cargo within the cilium is dependent on IFT. Proteins entering the cilium are assembled onto IFT trains consisting of multiple IFTB and IFTA repeating subcomplexes. These trains bind kinesin II for anterograde transport along the axoneme to the ciliary tip and also carry inactivated cytoplasmic dynein-2. At the ciliary tip, the complex re-assembles and dynein-2 activates to drive retrograde transport to the ciliary base. Kinesin-II passively diffuses back. IFTB primarily drives anterograde transport and IFTA primarily drives retrograde transport (Taschner and Lorentzen, 2016). (1) In most ciliated animal cells, the Golgi lies in close proximity to the basal body (Poole et al., 1997). (2) Collagen fibres have also been noted to interact with the ciliary membranes (Jensen et al., 2004). (B) The subunit and sub-complex composition of IFTA and IFTB particles for reference (Taschner and Lorentzen, 2016).

Primary cilia are microtubule-based protrusions at the cell surface that act as antenna, receiving and transducing extracellular signals from within the tissue and the environment (Satir et al., 2010). Their core structural component is a cylindrical ring of nine microtubule doublets called the axoneme, which extends from an adapted mother centriole (Ishikawa and Marshall, 2011). The axoneme is encased by the ciliary membrane, which anchors to the distal appendages of the mother centriole at the ciliary base (see Fig. 2). Although this membrane is continuous with the plasma membrane, it maintains a unique composition due to the presence of a proteinaceous transition zone at the ciliary base that blocks diffusion (Szymanska and Johnson, 2012). In many cell types, a significant portion of the cilium is embedded in the cell body. This creates a membrane invagination called the ciliary pocket which can act as an endocytic hotspot (Molla-Herman et al., 2010).

Membrane receptors and ion channels are particularly enriched on the ciliary membrane, consistent with a role in signal transduction. This enrichment, as well as cilium growth and maintenance, relies on the movement of proteins along the axoneme by a specialised intraflagellar transport (IFT) system (see Fig. 2; Bhogaraju et al., 2013). Within this system, two large protein sub-complexes, IFTA and IFTB, assemble into IFT trains at the base of the cilium and simultaneously bind cargo and microtubule-based motors to mediate transport. IFTB and kinesin-II primarily direct anterograde transport to the cilium tip (Zhu et al., 2021), while IFTA and dynein-II mediate retrograde transport back to the base (Vuolo et al., 2020).

## The cilium as a master signaller

Of all the inter-compartmental relationships addressed in this review, the role of the cilium on the ECM is the best studied. The impact of ciliary signalling on ECM production is multi-factoral, affecting tissue composition both indirectly, by regulating the differentiation of matrix-producing cells, and directly through transcriptional regulation and membrane dynamics. The literature on this topic is expansive, therefore this section highlights evidence pertinent to the current discussion, with a particular focus on the musculoskeletal system. For more detailed information please consult these dedicated reviews (Chinipardaz et al., 2022; Tao et al., 2020; Collins and Wann, 2020).

### Signalling and cell differentiation

The pathology of ciliopathies such as Jeune asphyxiating thoracic dystrophy and short rib polydactyly syndrome includes skeletal abnormalities (Handa et al., 2020). This suggests that skeletal tissues, which contain extensive amounts of ECM, are particularly sensitive to cilium loss, although extra-skeletal tissues are also often affected. This is perhaps unsurprising given the importance of ciliary signalling during skeletal development. Cilia and IFT are required for Hh signalling downstream of both Shh during limb patterning (Tickle and Towers, 2017; Zhang et al., 2003), and Ihh during endochondral ossification, the process by which chondrocytes lay down a cartilage-like template that matures into bone (St-Jacques et al., 1999; Haycraft et al., 2007). Ciliary platelet-derived growth factor (PDGF) signalling is also required for osteogenic differentiation of mesenchymal stem cells (Tummala et al., 2010), while Wnt signalling is important during cartilage development (Usami et al., 2016). Nonetheless, the requirement in these tissue for a rich ECM is also likely a factor. As load-bearing tissues, bone and cartilage are reliant on ciliary mechanotransduction for regulation, as described below. The susceptibility of these tissues to ciliary dysfunction therefore likely stems from the number of regulatory steps at which cilia are required.

Many studies have examined the contribution of specific components of the ciliary machinery during skeletal development (Yuan et al., 2015). The ablation of core ciliary machinery such as IFT80 (Yuan et al., 2016; Tao et al., 2019), IFT140 (Tao et al., 2019) and KIF3A (a kinesin-II subunit; Qiu et al., 2012) in osteoprogenitors has been shown to impair Hedgehog (Hh) signalling and osteoblast differentiation, leading to osteopenia, growth retardation and low bone mass. Similarly, loss of ciliary resident protein polycystin-1 impedes osteoblast differentiation (Xiao et al., 2006) and collagen production (Mangos et al., 2010). In cartilage, loss of IFT disrupts Hh and Wnt signalling, preventing chondrocyte differentiation in the growth plate (Wang et al., 2013) and impacting articular cartilage thickness and tidemark patterning (calcification) in post-natal joints (Rux et al., 2022; Coveney et al., 2022). The Evc protein, mutated in the skeletal ciliopathy Ellis van-Crevald syndrome, has also been shown to mediate Ihh signalling in both chondrocytes and osteoblasts to promote growth plate development (Pacheco et al., 2012; Ruiz-Perez et al., 2007). Cilia are therefore vital to the differentiation of mature, specialised ECM-secreting cells.

### Mechanotransduction

Almost all tissues are subject to mechanical stresses such as compression, tensile forces, and shear stress. To meet these demands, cells must be able to sense the biomechanical properties of their environment and adapt the ECM accordingly, a concept outlined in the ‘mechanostat’ model described by Frost (Frost, 2003). Increased mechanical loading promotes both bone and articular cartilage formation, while too little leads to ECM loss (Moore et al., 2018; Temiyasathit et al., 2012; Wong and Carter, 2003). Matrix stiffness can also direct the differentiation of mesenchymal stem cells towards neurons, myoblasts and osteoblasts (Engler et al., 2006; Gobaa et al., 2015).

In most tissues, mechano-sensation and mechano-transduction are at least in part conducted by primary cilia. Classically this is best studied in the context of ciliary bending in response to fluid flow in the kidney, however this is relevant to many tissues. For example, fluid flow during bone compression upregulates osteoblastic gene expression in an IFT88-dependent manner (Moore et al., 2018), although the signalling pathway used appears different to that in the kidney (Malone et al., 2007). Cilia also detect ECM deformations occurring in response to compressive and tensile force. Ultrastructural studies show a tight association between the ciliary membrane and collagen fibres in cartilage, with electron dense linkages forming at sites of contact (Poole et al., 1997; McGlashan et al., 2006; Jensen et al., 2004). Furthermore, integrins α2, α3 and β1, which link cell membranes to the ECM, have all been observed on the ciliary membrane (McGlashan et al., 2006). Cilia are therefore well placed to act as sensors of mechanical forces.

Repetitive activities, such as running, subject tissues to cyclic strain and trigger an upregulation of ECM production. In cartilage, this pathway is well elucidated. Upon the application of cyclic strain, ATP is released through hemichannels in the chondrocyte cell membrane. This induces a calcium influx that activates purinergic signalling through the P2 receptor and stimulates transcriptional changes in ECM genes (Chowdhury and Knight, 2006; Wann et al., 2012). In this case, cilia are dispensable for mechanosensation and ATP release, however activation of the purinergic pathway is IFT88-dependent (Wann et al., 2012). Cilia and the polycystins are also required for cyclic strain induced upregulation of aggrecan and collagen II, independent of ATP signalling (Thompson et al., 2021). Overall, loss of cilium-dependent mechanotransduction reduces the compressive modulus of cartilage (Irianto et al., 2014).

The mechanical environment can also impact ciliogenesis itself. Cyclic and static strain induce cilium shortening in chondroctyes and tendons respectively, courtesy of HDAC6-mediated resorption (Thompson et al., 2014; Rowson et al., 2018). Longer periods of strain also reduce cilium number (McGlashan et al., 2010). Conversely, the absence of strain in tendon causes cilium lengthening, likely due to ECM degradation (Rowson et al., 2016). Such responses may be tissue dependent, as load actually enhances ciliogenesis in the chicken growth plate (Rais et al., 2015). These adaptations to load are important, as cilium length and number determine mechanical sensitivity (Spasic and Jacobs, 2017; Khayyeri et al., 2015). This reciprocal feedback is disrupted in osteoarthritis, where the length and number of cilia increases with disease severity (McGlashan et al., 2008).

Besides HDAC6, the players and pathways connecting mechanical forces and ciliogenesis are largely unknown. One intriguing possibility, however, is that some of these inter-dependencies are transmitted by the transcriptional co-regulators YAP and TAZ. These proteins are targets of the Hippo kinase pathway which is activated in response to mechanical stimuli and integrin clustering at focal adhesions (Rausch and Hansen, 2020). Increased ECM stiffness correlates with increased phosphorylation and nuclear translocation of YAP/TAZ (Dupont et al., 2011). In turn, nuclear translocation of YAP/TAZ correlates with cilium disassembly (Rausch and Hansen, 2020). As with ciliary signalling, YAP/TAZ activity is crucial for coupling hMSC differentiation to substrate stiffness (Dupont et al., 2011). Furthermore, the ciliary proteins NPHP4 (Habbig et al., 2012), NPHP9 (Habbig et al., 2012), and several IFTB subunits (Peralta et al., 2020), have been shown to regulate YAP activity. Whether these observations represent remedial associations between ciliogenesis and ECM quality remains to be investigated.

### Membrane transport and matrix degradation

Ciliary signalling is important in regulating ECM degradation as well as production (see Collins and Wann, 2020). Gli proteins, the effectors of Hh signalling, can activate numerous genes involved in ECM turnover, including members of the ADAMTS, MMP, and TIMP families (Ali et al., 2019; Chang et al., 2012). Membrane dynamics at the cilium also orchestrate the removal of proteases from the extracellular space, protecting the ECM from degradation. Coveney et al. have shown that the cell surface receptor LRP-1 is organised on the periciliary membrane (Coveney et al., 2018). Here it recruits enzymes such as ADAMTS5 and MMP13, which degrade aggrecan and collagen respectively, to sites of clathrin mediated endocytosis in a process dependent on IFT. ADAMTS9, which degrades proteoglycans, is also internalised by LRP-1, and accumulates in Rab11 positive periciliary vesicles where it regulates ciliogenesis (Nandadasa et al., 2019). Altogether, this suggests the cilium is involved in organising an endocytic hotspot for ECM degradative enzymes (Coveney et al., 2018).

## The Golgi as a master builder

At the centre of the secretory pathway, the Golgi apparatus acts as both a manufacturing plant and logistics centre in regulating the quality and quantity of both ECM composition and ciliary signalling. Its function and regulation are therefore vital in co-ordinating tissue growth and homeostasis.

### Regulation of ECM chemistry

Arguably the most important Golgi function with respect to ECM secretion, beyond basic trafficking, is glycosylation. The addition of carbohydrates to proteins can increase their stability, modulate their physicochemical properties and activity, and regulate their secretion. Unlike proteins, polysaccharide chains are not templated and rely on the combinatorial use of multiple promiscuous and redundant enzymes. Regulation is achieved by controlling enzyme abundance and organisation across the secretory pathway in response to demand. Given the abundance of glycoproteins in the ECM, it is perhaps unsurprising that mutations in Golgi machinery frequently lead to connective tissue disorders (reviewed in Hellicar et al., 2022).

**Fig. 3.**
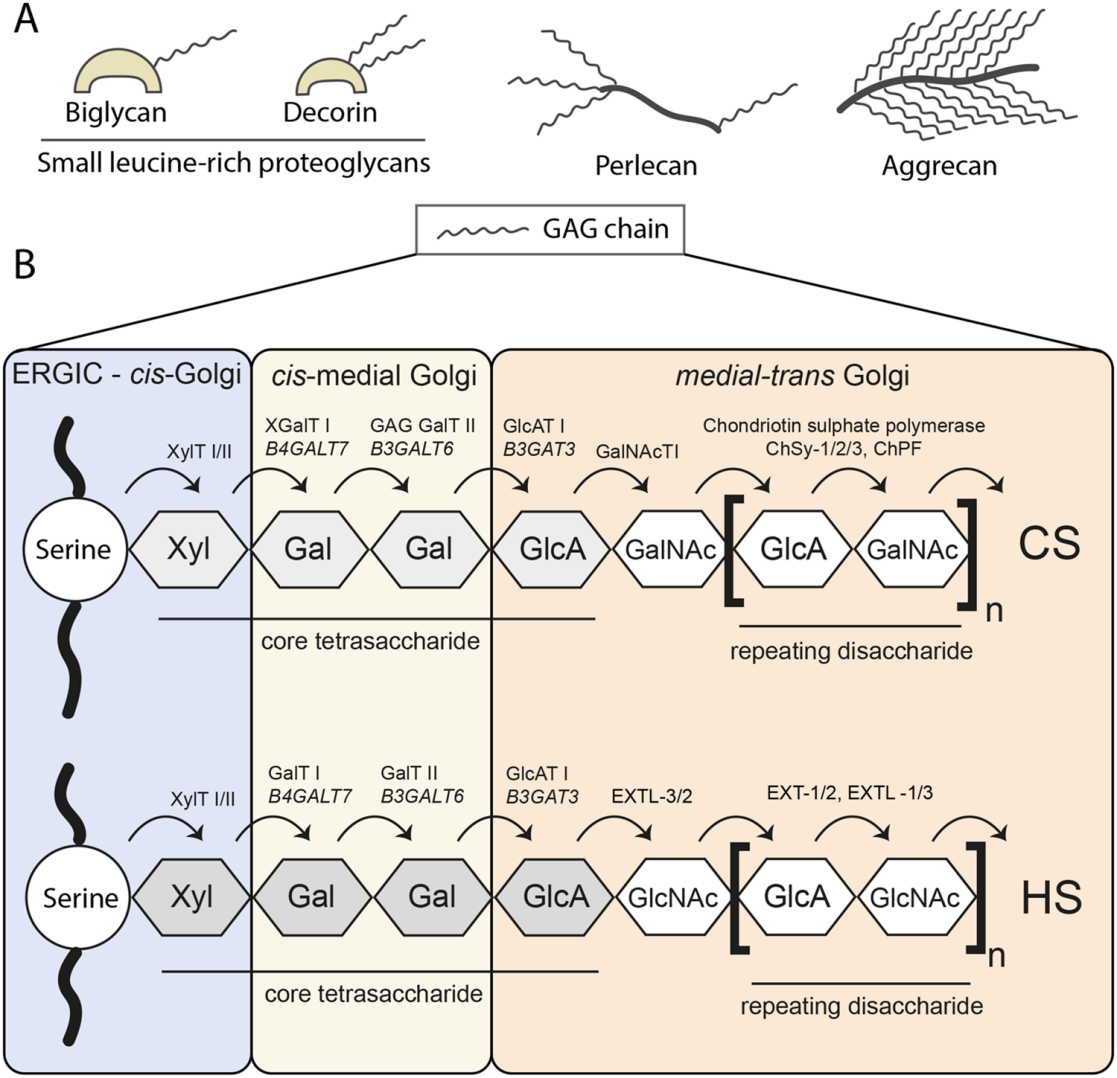
**GAG synthesis.** (A) Proteoglycans consist of a protein core and at least one large polysaccharide GAG chain. (B) There are four types of GAG chain, distinguished by their repeating disaccharide: chondroitin, (CS) dermatan, keratan and heparan sulphate (HS) (Silbert, 1982). The initiation of glycanation begins in the ER and early Golgi where xylose transferase I/II adds xylose (Xyl) to serine residues in the peptide chain (Vertel et al., 1993). The *cis-medial* Golgi enzymes β-1,4 galactosyltransferase 7 (XGalTI/B4GALT7) and β-1,3 galactosyltransferase 6 (GAG GalTII/B3GALT6) then work sequentially to add two galactose (Gal) residues to the xylose. Next, β-1,3-glucuronyltransferase 3 adds glucuronic acid (GlcA) in the *medial-trans* Golgi to complete the formation of the core tetrasaccharide common to most GAGs (Pedersen et al., 2000). From there, 100^+^ repeating disaccharide units are added to the core tetrasaccharide, as shown in the diagram. Dermatan sulphate is created through the epimerisation of GlcA to IdoA in the chondroitin sulphate chain (Tykesson et al., 2018). Keratan sulphate is more complex and not discussed here. All chains are sulphated in the *medial-trans* Golgi concurrent with chain elongation (Sugumaran and Silbert, 1990). The sequential nature in which GAGs are built is reflected in the organisation of their enzymes across the Golgi stack and provides a good example of enzyme compartmentalisation in practice. Adapted from Hellicar et al., 2022.

Proteoglycans are particularly susceptible to Golgi dysfunction. Members of this family consist of a protein core to which one or more glycosaminoglycan chains (GAG) are covalently attached. The assembly of GAG chains is initialised by the construction of a core tetrasaccharide on a serine residue, followed by the addition of >100 repeating disaccharide units, which are then sulphated (Fig. 3, Prydz, 2015; Silbert, 1982). This sequential assembly is facilitated by the sequential distribution of enzymes across the Golgi stack and is therefore sensitive to alterations in intra-Golgi trafficking (Fig. 3). Loss or overexpression of the COPI adapter GOLPH3, for example, disrupts the recycling of exostosins (EXT genes in Fig. 3) leading to incomplete glycation of heparan sulphate (Chang et al., 2013). Proteoglycan production is also impaired following loss of the COG complex, which tethers retrograde recycling vesicles (Adusumalli et al., 2021). Meanwhile, loss of the stacking proteins GRASP55 and GRASP65 disrupts enzyme abundance, increasing heparan sulphate synthesis at the expense of chondroitin sulphate (Ahat et al., 2022).

The presence of GAG chains is functionally important within tissue. For example, the small leucine-rich proteoglycans (SLRPs) decorin and biglycan both bind to TGF-β in a GAG dependent manner to regulate signalling (Hildebrand et al., 1994), whilst heparan sulphate can bind to a number of morphogens such as wingless, Decapentaplegic and Wnt in Drosophila to regulate developmental patterning (Nybakken and Perrimon, 2002). The GAG chains of SLRPs may also co-ordinate mineral deposition in bone (Waddington et al., 2003) and impact collagen fibrillogenesis (Kuc and Scott, 1997; Ruhland et al., 2007; Maccarana et al., 2009). Golgi regulation of proteoglycan synthesis is therefore vital to many diverse processes.

The chemistry of the Golgi more generally can affect ECM production. Mutations in the V-type H^+^ ATPase impair Golgi acidification and therefore glycosylation and trafficking, and lead to a range of diseases associated with wrinkled skin and bone abnormalities (Van Damme et al., 2020; Kornak et al., 2008). Meanwhile, a failure to import copper following mutation of the TGN resident ATP7A copper ion pump impedes the cuproenzyme lysyl oxidase, required to initiate collagen and elastin crosslinking (Skjorringe et al., 2017; Shanbhag et al., 2019). Depending on the severity of mutation this leads to the connective tissue disorders Menkes disease and occipital horn syndrome (Skjorringe et al., 2017; Caicedo-Herrera et al., 2018; Moller et al., 2000; Yasmeen et al., 2014).

### Regulation of transcription

The Golgi is also involved in ECM regulation at the level of transcription. The ER-resident, type II transmembrane proteins CREB3L1/OASIS and CREB3L2/BBF2H7 are cleaved in the Golgi by site-1 and site-2 proteases (S1P and S2P) in response to stimuli. Proteolysis liberates an N-terminal fragment that translocates to the nucleus to bind to cAMP response elements in specific genes (Sampieri et al., 2019). CREB3L1 is particularly highly expressed in osteoblasts in which cleavage is stimulated by osteogenic signals such as TGFβ (Chen et al., 2014) and BMP2 (Murakami et al., 2009). This activates expression of collagen type I and the COPII component Sec24D (Keller et al., 2018). Loss of CREB3L1 function in mice and humans leads to osteogenesis imperfecta with affected individuals showing growth retardation and brittle bones (Keller et al., 2018; Murakami et al., 2009). CREB3L2 on the other hand activates the expression of the COPII machinery in chondrocytes and its deletion in animal models results in skeletal dysplasia and craniofacial defects (Saito et al., 2014; Ishikawa et al., 2017). In both cases, a failure to upregulate COPII leads to ER retention of procollagen.

Mutations in the proteases themselves lead to similar outcomes, with S1P loss causing skeletal dysplasia and S2P loss linked to osteogenesis imperfecta. Patients with impaired S1P function also experience excessive ECM degradation due to the aberrant secretion of lysosomal enzymes (Kondo et al., 2018), while the collagen of patients lacking S2P activity is poorly cross-linked due to impaired lysine hydroxylation (Lindert et al., 2016). These additional disruptions to the ECM presumably result from a failure to cleave substrates other than CREB3L1/2. The Golgi therefore acts as a central hub for ECM production, working not only to manufacture complex glycoproteins but also to monitor demand and adapt output in a responsive manner.

### The modification and transport of ciliary proteins

Most ciliary membrane proteins pass through the Golgi apparatus where they undergo modification in a similar manner to ECM proteins. Glycosylation again appears particularly important, since aberrant glycosylation of specific ciliary proteins, such as the polycystins, is linked to disease (Inoue et al., 2014). Defects in the glycosylation of proteins regulating ciliogenesis are also detrimental. For example, loss of the glycosyltransferase GALNT11, and a subsequent failure to glycosylate Notch1, results in the dysregulation of sensory versus motile cilia formation during development (Boskovski et al., 2013).

More global defects in glycosylation pathways can also impact ciliary function. In 2017, Kane et al. found that mutation in the centrosomal protein OFD1 in Joubert syndrome type 10 patients impairs both protein sialylation, which occurs in the *trans* Golgi, and ciliogenesis (Kane et al., 2017). In control cells, sialic acid modifications are detectable in the cilium and treatment with neuraminidase, which digests sialic acid, reduces cilium length and prevalence. This demonstrates Golgi resident modifications are required for cilium maintenance, and that a single gene can affect both Golgi and ciliary function, as discussed in more detail below.

Despite not transiting the secretory pathway lumen, some soluble ciliary proteins are still dependent on the Golgi for modification and ciliary targeting. The Golgi resident palmitoyl-transferase DHHC-21, which presents its catalytic site on the cytoplasmic face of Golgi membranes, has been found to palmitoylate N-terminally myristoylated ciliary proteins (Kumeta et al., 2018). This generates a dual lipidation-coupled ciliary targeting signal to promote translocation to the cilium. Palmitoylation also appears important for protein stability and circumventing size-exclusion at the transition zone (Kumeta et al., 2018). One DHHC-21 client, Retinitis pigmentosa 2 protein, is itself also required for vesicular traffic between the Golgi and cilium (Evans et al., 2010; Kane et al., 2017).

### Where ECM and ciliary proteins meet

In addition to ciliary-resident proteins, secreted signalling molecules that activate receptors on the ciliary membrane, such as Hh, must also pass through the Golgi. Here, an intriguing interplay between ECM protein glycosylation and Hh has recently been proposed (Tang et al., 2022). Hh proteins contain a Cardin-Weintraub (CW) sequence in their N-terminus, a motif that is known to mediate interactions with heparan GAGs. Tang et al. found that the CW motif of sonic hedgehog (Shh) binds SURF4 in the ER to mediate ER-Golgi transport (Tang et al., 2022). Once in the Golgi, further anterograde transport requires the presence of GAGs to outcompete SURF4 binding at the CW motif and release Shh for Golgi exit. This suggests a mechanism by which Hh signalling may be co-ordinated with proteoglycan production within the secretory pathway.

### IFT20 – master of all trades

One protein that has been widely recognised as a bridge between Golgi and ciliary function is the IFT-B subunit IFT20 (Follit et al., 2006; Follit et al., 2008). As a core part of the IFT machinery, IFT20 localises to the cilium (Follit et al., 2006) where it connects IFT particles to kinesin II (Baker et al., 2003) and regulates receptor signalling (Schmid et al., 2018). Importantly, it is also recruited to the Golgi by the golgin GMAP210, as discussed below (Follit et al., 2006; Follit et al., 2008; Noda et al., 2016). Here it is suggested to support transport through (Nishita et al., 2017) and out of the Golgi (Keady et al., 2011; Finetti et al., 2020; Yang et al., 2021). During cancer cell invasion it can also stimulate microtubule nucleation on Golgi membranes to promote polarised secretion of MMPs for ECM degradation (Nishita et al., 2017).

Unsurprisingly given its residence at such prime cellular real estate, IFT20 function is vital to skeletal development. Conditional knockout (cKO) of *Ift20* in cranial neural crest cells in mice leads to severe craniofacial malformation that is peri-natal lethal (Noda et al., 2016). Osteoblast differentiation in these animals proceeds normally, but proliferation and survival are affected by the loss of cilium mediated PDGF signalling. Consequently, calcium deposition is patchy and osteoblasts produce thin, discontinuous collagen fibrils (Noda et al., 2016; Yamaguchi et al., 2021), likely due to impaired ER-Golgi transport of procollagen type I (Noda et al., 2016). Post-natal cKO of *Ift20* in osteoblasts has a similar effect (Yamaguchi et al., 2020; Lim et al., 2020), however, lysyl hydroxylation of procollagen, and therefore collagen cross-linking, is also disrupted due to increased expression of the lysyl hydroxylase 2 chaperone, FKBP65 (Yamaguchi et al., 2020). Finally, cKO of *Ift20* in condylar cartilage results in fewer, smaller chondrocytes and loss of Hh signalling-induced collagen type X expression (Kitami et al., 2019). Overall, IFT20 is required for ciliary signalling, Golgi function, and collagen modification and secretion during bone and cartilage formation, putting it at the heart of a Golgi-cilium-ECM continuum, a concept first described by Poole et al. (2012).

## Cross-regulation of organelle organisation

The close apposition of the Golgi apparatus and the centrosome is an almost universal feature of mono-ciliated vertebrate cells. The importance of this relationship, however, has been challenging to study (Rios, 2014). AKAP450 is a Golgi and centrosome localised protein that nucleates microtubules on Golgi membranes (Rivero et al., 2009). In 2011, Hurtado et al. (2011) found that disrupting AKAP450 function by expressing a dominant negative N-terminal fragment, partially uncouples Golgi and centrosome position (Rivero et al., 2009) and prevents microtubule nucleation on Golgi membranes. Intriguingly, this also blocks ciliogenesis. Contrary to this, Tormanen et al. found that knockout of GM130 uncouples the centrosome and Golgi without impacting ciliogenesis (Tormanen et al., 2019). Disparities between the studies may stem from the extent to which AKAP450 function is disrupted, since GM130 knockout only caused a partial loss of AKAP450 without disrupting Golgi microtubules. The formation of Golgi-nucleated microtubules may therefore be more important for ciliogenesis than organelle proximity.

The relationship between the ECM and cilium orientation has also been challenging to dissect. In tendon, which is mechanically loaded in a single direction, collagen fibres and the ciliary axoneme align parallel to the direction of stretch (Donnelly et al., 2010) whereas cilium orientation is random in isolated tenocytes (Rowson et al., 2018). It remains unclear if this alignment in tissue is determined passively by the structural constraints imposed by an organised ECM, or more actively directed by mechanotransduction. Chondrocyte cilium orientation is also particularly well organised in cartilage, with chondrocyte cilia orientated away from the articular surface in the superficial zone, and between two cells or on the lateral surface in the middle and deep zone (McGlashan et al., 2008). Interestingly, orientation is more consistent in load-bearing regions of cartilage (Farnum and Wilsman, 2011). Mechanotransduction may therefore be relevant in some tissues.

## The golgins caught at the intersection

The golgins represent a key family of membrane tethers on the Golgi surface, responsible for facilitating interactions between Golgi membranes and transport carriers. They are anchored to Golgi cisternae via their C-terminal domain and project long-coiled coil domains harbouring vesicle binding sites out into the cytosol. The restriction of each golgin to certain cisternae, combined with their selective affinity for particular vesicle subsets, confers tethering and fusion specificity (Wong and Munro, 2014). Like *OFD1* and *IFT20*, loss of some golgins has been shown to simultaneously affect the Golgi, cilium, and ECM further illustrating the interdependence of these compartments.

### GMAP210

Perhaps the best example of a gene at the centre of the Golgi-cilium-ECM continuum is *TRIP11*, which encodes the cis-Golgi resident golgin, GMAP210. In humans, null mutations in *TRIP11* are causative of achondrogenesis type 2A (ACG2A), while hypomorphic mutations have been identified in cases of odontochondrodysplasia (ODCD) (Upadhyai et al., 2021; Medina et al., 2020; Del Pino et al., 2021; Wehrle et al., 2019). Both diseases are characterised by skeletal dysplasia and poor ECM production, including shortened long bones, craniofacial defects, and delayed or impaired mineralisation (Medina et al., 2020; Upadhyai et al., 2021; Wehrle et al., 2019). The pathogenesis of these diseases has been studied in mice, in which *Trip11* deletion largely phenocopies human pathology (Bird et al., 2018; Follit et al., 2008; Smits et al., 2010). These reports show that the skeletal dysplasia observed in both species is largely due to impaired chondrocyte differentiation during endochondral ossification (Bird et al., 2018). Meanwhile, the craniofacial defects likely result from osteoblast dysfunction during intramembranous ossification (Yamaguchi et al., 2021).

The chondrogenesis defects observed in *Trip11* mutants resemble those frequently observed following loss of ciliary function. Interestingly, many patients with ODCD acquire additional ciliopathy-like phenotypes such as cystic kidneys and macrocephaly (Wehrle et al., 2019). The role of GMAP210 in ciliogenesis has therefore become an active focus of study. Upadhyai et al. found that ciliogenesis is impaired in human patient fibroblasts (Upadhyai et al., 2021) and cilium length is reduced in both mouse and human mutant cells (Upadhyai et al., 2021; Follit et al., 2008). Perhaps most crucially, GMAP210 was found to be required for IFT20 localisation to the Golgi, directly connecting the golgin to ciliary function (Wehrle et al., 2019; Follit et al., 2008, 2006).

The IFT20-GMAP210 complex seems to be particularly important during intramembranous ossification in the skull, a process of bone formation in which woven bone is laid down directly by osteoblasts (Galea et al., 2021). Loss of either IFT20 or GMAP210 causes similar craniofacial defects and reductions in collagen type I secretion, although only *Ift20* mutations affect osteogenesis (Noda et al., 2016; Yamaguchi et al., 2021). Knockout of both genes increases the severity of the secretion defect (Yamaguchi et al., 2021). Endochondral ossification also depends on both genes, but they appear to act at different stages, with IFT20 function required in the mesodermal lineage and GMAP210 in chondrocytes (Yamaguchi et al., 2022).

In addition to ciliogenesis and cellular differentiation, loss of GMAP210 impacts ECM secretion and Golgi morphology. (Wehrle et al., 2019). Collagen type I secretion and the production of highly glycanated proteoglycans is impaired in fibroblasts from ODCD and ACG1A patients, although bulk secretion is only disrupted in the latter. Perlecan also seems to be particularly sensitive to GMAP210 function as it is retained in the ER in *Trip11^-/−^* murine chondrocytes (Bird et al., 2018; Smits et al., 2010). Golgi structure itself is impacted in a cell-type-specific manner. Chondrocytes from prenatal knockout animals contain expanded ER and Golgi compartments (Bird et al., 2018; Smits et al., 2010), whereas other cell types show variable Golgi phenotypes ranging from abnormal stacking in osteoblasts, compacted and vesiculated membranes in fibroblasts (Wehrle et al., 2019), and normal morphology in osteoclasts (Bird et al., 2018). A similar situation arises upon loss of IFT20. IFT20 depletion in RPE cells (Follit et al., 2006) and KO in mouse osteoblasts (Noda et al., 2016) has no effect on Golgi structure, while loss affects Golgi size in condylar chondrocytes (Kitami et al., 2019) and the integrity of the Golgi ribbon in the SaOS2 osteosarcoma line (Nishita et al., 2017). Considering the close relationship between IFT20 and GMAP210, dissecting the contribution of trafficking versus ciliary function in the pathogenesis of *TRIP11*-dependent ACG1A and ODCD poses a challenge.

### Giantin

A second golgin implicated in both ciliogenesis and Golgi function is the *cis*-Golgi tether giantin, encoded by *GOLGB1*. Interestingly, loss of giantin function manifests differently between species but always impacts the skeleton. *Golgb1* mutant rats share many phenotypes with *Trip11* mutant mice, including shortened ribs and long bones, craniofacial defects, impaired chondrocyte differentiation, and attenuated Ihh signalling (Katayama et al., 2011). Loss of giantin in mice is also neonatal lethal, although the phenotype is milder, with individuals appearing grossly normal apart from a cleft palette (Lan et al., 2016). Zebrafish mutants show a mild developmental delay (Bergen et al., 2017), accumulate fractures, and experience abnormal collagen expression and mineralisation during fracture repair (Stevenson et al., 2021). Their soft tissue also contains ectopic mineral deposits, like those seen in tumoral calcinosis (Stevenson et al., 2017). To date no mutations in *GOLGB1* have been reported in humans.

Most studies agree that loss of giantin has little impact on Golgi morphology (Stevenson et al., 2017; Puthenveedu and Linstedt, 2001; Koreishi et al., 2013), although it may reduce cisternal connectivity (Satoh et al., 2019; Stevenson et al., 2017). It does however impact the enzymatic composition of the Golgi. Depletion of giantin with siRNA affects protein sialylation (Koreishi et al., 2013) and the trafficking of specific enzymes (Petrosyan et al., 2014), while knockout leads to more global changes in gene expression (Stevenson et al., 2017; Lan et al., 2016; Kikukawa et al., 1990). Hyaluronic acid and GAG production is particularly altered in mutant rats and mice, although other glycosylation pathways may be affected in a tissue specific manner (Katayama et al., 2018; Lan et al., 2016). In zebrafish, only the downregulation of the glycosyltransferase GALNT3 has been investigated (Stevenson et al., 2017).

ECM production is also affected by loss of giantin. In mutant rats, collagen type XI expression is upregulated, whilst aggrecan and link protein expression is low and uneven in cartilage but ectopically upregulated elsewhere (Katayama et al., 2018). Studies in cultured cell lines have also shown that giantin is required for intracellular N-terminal processing of procollagen type I (Stevenson et al., 2021). Giantin is therefore pivotal in regulating multiple aspects of ECM composition.

In addition to its role in manufacturing ECM glycoproteins, giantin regulates ciliogenesis. Knockdown of giantin in multiple cell lines and in zebrafish larvae results in a reduction in both cilia number and length (Asante et al., 2013; Bergen et al., 2017). Complete gene knockout only affects cilium length (Bergen et al., 2017; Stevenson et al., 2017), however this may be due to compensatory upregulation of RCAN2 (Stevenson et al., 2018). The cilia that are produced in these models have a bulbous tip, indicative of retrograde IFT defects (Bergen et al., 2017). Consistent with this, dynein-2 localisation to the ciliary base is reduced in giantin knockdown cells (Asante et al., 2013). Dynein-2 mutations in humans are associated with skeletal ciliopathies (Vuolo et al., 2020). The link between cilium function and ECM production in giantin models is yet to be investigated.

### GORAB

Another golgin implicated in connective tissue disorders and ECM secretion is GORAB. Mutations in *GORAB* have been identified in cases of geroderma osteodysplastica (GO), a progeroid disorder characterised by wrinkled skin, joint laxity, osteoporosis, and growth retardation (Al-Dosari and Alkuraya, 2009; Hennies et al., 2008). Unlike GMAP210 and giantin, GORAB resides on *trans*-Golgi membranes where it recruits COPI to sites of vesicle formation by binding the adapter Scyl1 (Egerer et al., 2015; Witkos et al., 2019). GO patient and *Gorab^null^* mouse fibroblasts have a largely normal Golgi structure (Hennies et al., 2008; Liu et al., 2016), with some distention of the *trans*-Golgi (Witkos et al., 2019), however they produce reduced amounts of terminally sialylated glycans due to defective recycling of Golgi enzymes (Witkos et al., 2019). Anterograde transport of decorin through the Golgi is also attenuated, although global secretion is unaffected (Chan et al., 2018).

The impact of GORAB loss of function on ECM secretion has again been studied in mice. Whole body *Gorab^null^* mutations are neonatal lethal due to a reduction in alveolar space, but craniofacial defects can be observed (Liu et al., 2016) as well as abnormal collagen deposition in the dermis (Chan et al., 2018). Conditional knockout in the mesenchyme lineage emulates post-natal human pathology more closely (Chan et al., 2018; Yang et al., 2017). In these animals, bone growth is retarded, mineral apposition is slow, and terminal osteocyte differentiation is compromised (Chan et al., 2018; Yang et al., 2017). Collagen fibrillogenesis and alignment within the bone is also impaired, and the total amount of GAGs is highly diminished, suggesting defects in glycanation (Chan et al., 2018).

The link between GORAB and ciliary function is more tenuous than for the other golgins discussed. However, there are suggestions they interact. In *Drosophila*, monomeric Gorab localises to centrioles (Fatalska et al., 2021; Kovacs et al., 2018), and the cilia of *gorab^null^* sensory neurons have disorganised ciliary rootlets and disrupted ninefold radial symmetry (Kovacs et al., 2018). In these studies, Gorab function in Golgi trafficking and ciliogenesis can be separated suggesting the compartments are not directly interacting. Fly and human GORAB proteins share 70% similarity, however it remains unclear to what extent these findings extrapolate to vertebrate systems.

A study of perhaps clearer significance for vertebrate systems is that of Liu et al., performed in *Gorab*^null^ mice. At E18.5 they found that the dermis of mutant animals possessed fewer hair follicles due to impaired morphogenesis. This was associated with the loss of cilia in dermal mesenchymal cells and the subsequent disruption to Hh signalling (Liu et al., 2016). GORAB function is therefore required for ciliogenesis in at least in one cell type. Due to the lethality of the whole organism knockout, ciliogenesis has not been looked at post-natally in other tissues or at later developmental stages. Whether this observation has wider implications beyond hair follicle morphogenesis therefore remains to be determined.

## Perspectives

**Fig. 4.**
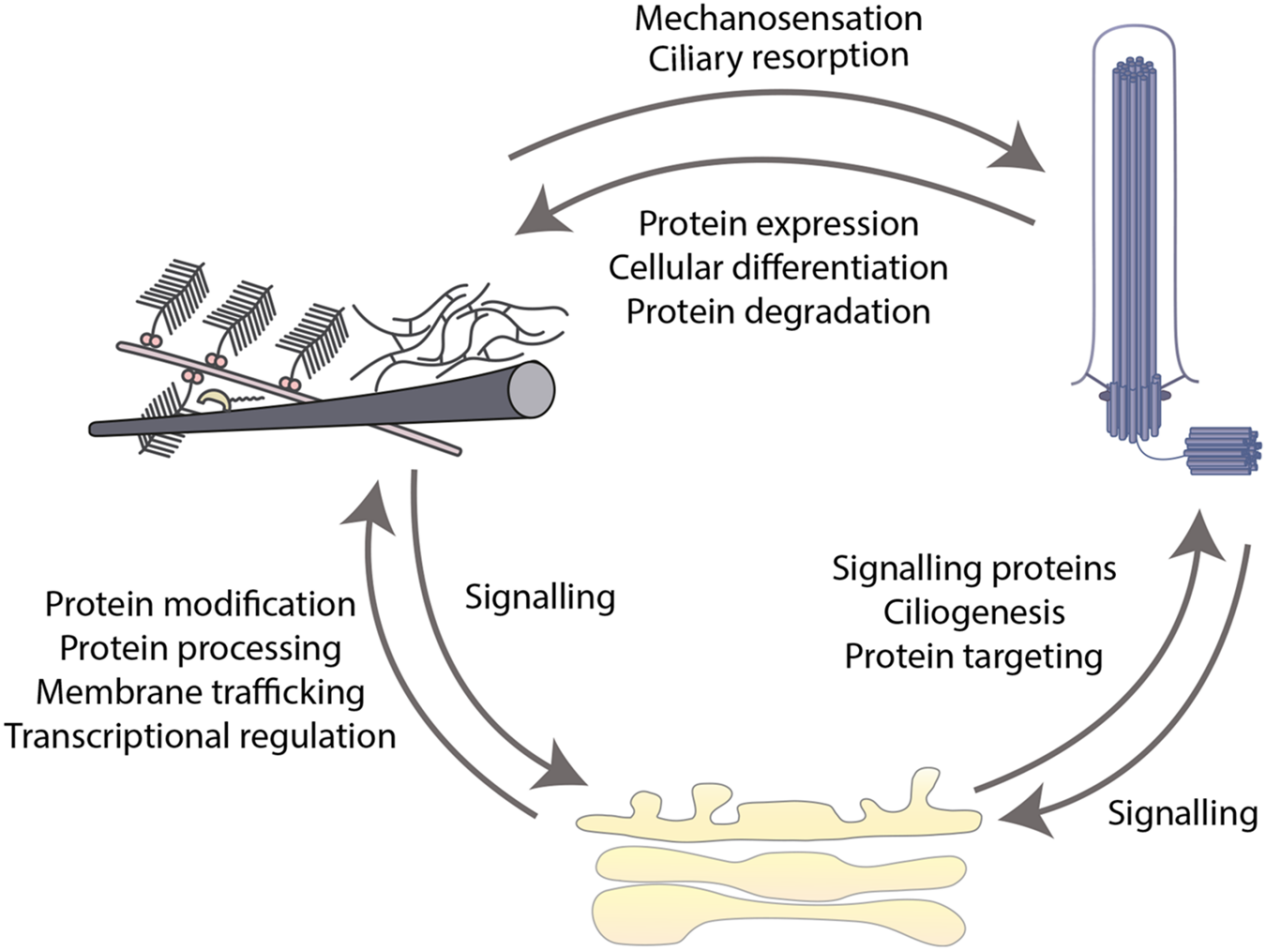
**The three-way interplay between the cilium, Golgi, and ECM.** A diagram to show the key relationships between the cilium, Golgi, and ECM.

The studies discussed here provide strong evidence of a functional continuum between the Golgi, cilium, and matrix, which extends beyond mere spatial proximity (Fig. 4). Given the number of genes already identified that affect all three compartments, the inter-connectedness of this system must be considered in future studies when dissecting cause and effect. Disruption could arise at any point and would quickly exacerbate as defects in one compartment feedback on the others. Many authors, including myself, have argued that loss-of-function in genes like the golgins most heavily impact the skeletal system due to the high secretory load in these tissues. While this may be true to some extent, perhaps it is time to consider that the sensitivity of the skeletal system may stem from its combined reliance on both Golgi output and ciliary signalling. Deciphering this interplay in the future is likely to require a broader approach combining *in vivo* and *in vitro* work across systems rather than studying these in isolation.
